# Influence of Exhaust System Setup on Working Zone Pollution by Dust during Sawing of Particleboards

**DOI:** 10.3390/ijerph17103626

**Published:** 2020-05-21

**Authors:** Bartosz Pałubicki, Luďka Hlásková, Tomasz Rogoziński

**Affiliations:** 1 Department of Woodworking Machines and Fundamentals of Machine Design, Faculty of Wood Technology, Poznań University of Life Sciences, ul. Wojska Polskiego 38-42, 60-637 Poznań, Poland; bpalubic@up.poznan.pl; 2 Department of Wood Science and Technology, Faculty of Forestry and Wood Technology, Mendel University in Brno, Zemědělská 3, 61300 Brno, Czech Republic; ludka.hlaskova@mendelu.cz; 3 Department of Furniture Design, Faculty of Wood Technology, Poznań University of Life Sciences, ul. Wojska Polskiego 38-42, 60-637 Poznań, Poland

**Keywords:** wood dust, dust emission, sawing, particleboard, dust exhaust system

## Abstract

Air pollution by wood dust in furniture production sites is an important hygiene issue. The dust is created by all types of wood and wood-based material machining, and its concentration in the working zone surrounding the machining stand depends on the effectiveness of the dust exhaust system. In present research, three setups of the dust extraction system for a conventional table sawing machine are considered while machining particleboards. The results showed a high impact of the exhaust system connection setup on the dust concentration in the air surrounding the sawing machine work stand. The use of both main and auxiliary sawdust extraction connectors together ensured the highest clearness of the air, with only 0.5 mg/m^3^ of dust concentration. Closing the upper hood leads to a concentration five times higher, while disconnecting it results in a ten times higher dust content. The finest dust particles (<1 µm), however, are the most numerous in the case of closing the hood.

## 1. Introduction

Air dustiness in furniture production is the result of the creation of fine waste in the form of dust particles during woodworking. The amount of dust depends on the type of processed material, the method of processing, and the parameters used in processing. In principle, the machining of all wood materials used, including solid wood, wood materials, and thermally modified wood, is a source of air dustiness. Palmqvist and Gustafsson [1] concluded that different species of wood generate different levels of dust. Their experiments showed that the MDF (medium-density fibreboard) planing and milling creates up to six times as much dust as pine wood machining. Beech wood is a source about 50% more dust than pine. In a study on sawing wood and wood-based materials, Fujimoto et al. [2] states that the mass concentration of respirable dust from particleboard and tropical hardwood plywood was higher than from sogi wood (*Cryptomeria japonica)*, softwood plywood, and MDF. In case of thermally modified wood, the modification temperature has an influence on fine dust created during machining [3,4].

Sawing is a processing method used in all technological processes of wood products. Unfortunately, it is also the cause of significant air dustiness inside the production rooms [5]. Baran and Teul [6], as a result of researching over 1000 employees from the wood processing industry of nine Polish woodworking plants, recognized the positions of circular and band saws as the most threatening for the exposure of employees to air pollution, right after sanding stands.

Kos et al. [7], in research on the impact of the type and parameters of wood material processing on workplace dustiness, found that the dustiness of air sampled at belt sanders and circular saws significantly exceeded the assumed limit values. This research was carried out according to Croatian regulations. The limits in force then for the mass concentration for oak and beech wood respirable dust was 1 mg/m^3^, and for total dust was 3 mg/m^3^. The limit of 5 mg/m^3^ for inhalable hardwood dust is now valid, according to the European Council Directive 1999/38/EC on the protection of workers from the risks related to exposure to carcinogens at work and extending it to mutagens. It was also proved that the low average chip thickness causes an increase in the mass concentration of respirable particles near the working machines. These results were confirmed in the paper on dust emission and occupational wood dust exposure in the Croatian work industry by Čavlović et al. [8].

Chung et al. [9], in their study on dust emission during the sawing and sanding of MDF pine and oak wood, have stated that there is no significant difference in the quantity of dust created during sawing these materials. Also, the size distributions of these dusts were similar. However, further sawdust processing, including grinding and screening for sorting purposes, is much more hazardous in the aspect of air dustiness than sawdust creation during sawing. Liou et al. [10] found a more than four times higher total concentration of sawdust at sanding than at sawing in a Taiwanese woodworking plant processing different native wood species.

Many epidemiological studies on the adverse health effects associated with occupational exposure to wood dust list several diseases occurring in exposed workers. These are generally diseases of the human respiratory tract and skin. It has been proven that exposure to wood dust increases the risk of chronic lung disease, chronic bronchitis, nose and paranasal sinus cancer, mutagenic and genotoxic effects, eye and skin irritation, and allergic reactions. The allergic reactions, as well as occupational asthma, may be caused by bioactive compounds present in wood dust and the biological fraction (fungi and bacteria and their metabolites) [11,12,13,14,15,16,17,18,19,20,21]. The most important technical means to reduce this risk is the proper use of dust extraction systems. This also applies to circular saws. As Top [22] concluded, the lack of connection of the upper extraction hood to the circular saw causes insufficient removal of fine and light dust particles.

Incorrect positioning of the circular table saw’s suction hoods and pipes carries the risk of increased air dustiness. This results from the fact that the sawdust stream is clearly oriented, since the rotational motion of the saw and the exhausted air velocity vector should have a direction consistent with the natural movement of sawdust. New technical solutions regarding the construction of suction hoods in saw machines were presented by Wieloch and Mostowski [23]. Lack of care in this regard, and the resulting increased air dustiness in furniture factories, have been stated by Scheeper et al. [24]. Therefore, the purpose of the current research was to experimentally study the influence of various ways of connecting the saw’s suction hoods on the dustiness of the surrounding air.

## 2. Materials and Methods

Commercially available, melamine-coated (non-structured), three-layered, 18 mm thickness particleboard panels, produced by the Kronospan company, were used as a material for the experiments. Edges of the board sheets were trimmed before the experiment to remove loose structure material. The average density of the remaining panels was 649 kg/m^3^.

A K700 (Felder, Austria) formatting circular saw with a nominal spindle rotational speed of 4800 rpm was used in the tests. The average feed speed achieved with use of pneumatic actuator was 12 m/min. The tool used for the research was the saw with a diameter of 300 mm (Pilana, Czech Republic), kerf thickness 3.2 mm, and body thickness 2.2 mm, equipped with 96 teeth grouped into trapezoidal-straight pairs. The feed per tooth was therefore 0.026 mm.

The sawing machine was equipped with two sawdust extraction hoods: a main one in the machine body under the table, with a connector diameter of 120 mm, and an auxiliary one in the upper cover (hood) of saw blade, with a connection diameter of 80 mm.

**Figure 1:**
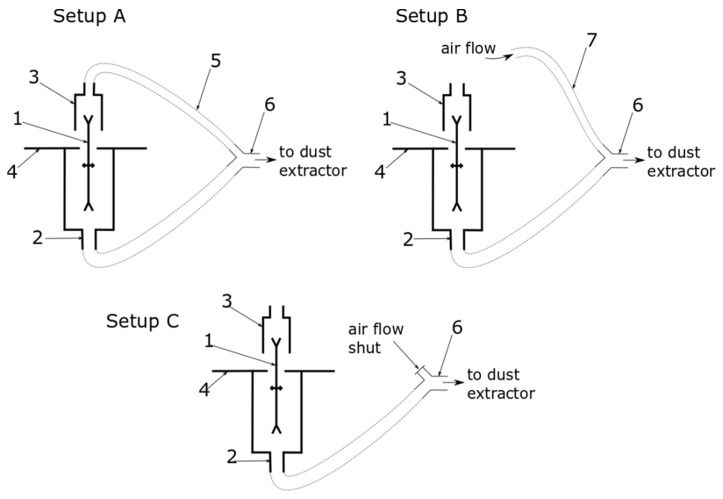
Experimental setup cases. 1: circular saw, 2: main suction connector, 3: saw hood with auxiliary dust suction connector, 4: saw table, 5: flexible pipe (hose), 6: double splitter pipe connector, 7: disconnected flexible pipe (hose).

Chip removal was carried out using a mobile dust extractor type AF14 (Felder, Austria) with a rated air flow of 2350 m^3^/h and a diameter of the connection tube 140 mm. An additional double splitter with 120 mm and 80 mm inlet diameters and a 140 mm outlet diameter was used. Thanks to this, it was possible to test three extraction connection setup options (Table 1, Figure 1). Both saws’ suction connectors were attached to the extractor with 2.5 m long flexible tubes. The fan of the dust extractor operated at a constant rotational speed, and the changes in resistance in the connected pipes caused changes in its output in accordance with the fan flow characteristics. The extraction parameters recommended by the machine manufacturer [25], together with the actual values measured during the experiment, are presented in Table 2. This measurement was made using a digital differential manometer CMR 10, (ZAM Kęty, Poland) connected with Prandlt tube placed in the extraction pipes.

The duration of the single experiment with one setup was 50 min. During this time, a 150 m length of cut was made (300 passes of 0.5 m each). The level of air dust surrounding the saw was measured. To measure air dustiness, a laser particle counter (LPC) type HR5025A (Pacific Scientific, United States), whose operation is based on the analysis of laser light scattering, was used. The measurement range of the counter included eight channels with the dimensional limits of 0.5, 1, 2, 3, 5, 10, 15, and 25 μm. The air flow rate of the counter was 0.0283169 m^3^/min. For each setup, each measurement, and each dimensional *i*-range, the number of dust particles found were recalculated by the LPC with respect to 1 m^3^ of air (*n_i_*: the number concentration of dust). Samples of the air were taken using the isokinetic probe, placed 0.4 m over the saw table and at a 0.5 m distance from saw axis. The sampling time was set to 30 s, and the delay time between successive measurements was also 30 s. In the processing time for each setup of the extraction system, 50 measurements were carried out. The result of the measurement was recorded as the count number, and the mass concentration had to be further calculated. The mass concentration (*C_i_*) of dust particles from the *i*-range and the total mass concentration *C* in the air surrounding the saw machine were calculated using the data obtained by the particle counter, as follows:

(1) C=∑i=18Ci [mgm3]

(2) Ci=n¯i·πd¯i36·ρ, [mgm3],

where *n_i_* is the average number concentration of dust particles measured on the *i*-channel of the counter (pcs/m^3^), *d_i_* is the average particle size of the *i*-channel (mm), and *ρ* is the density of the wood substance (1.5 mg/mm^3^).

## 3. Results and Discussion

**Figure 2:**
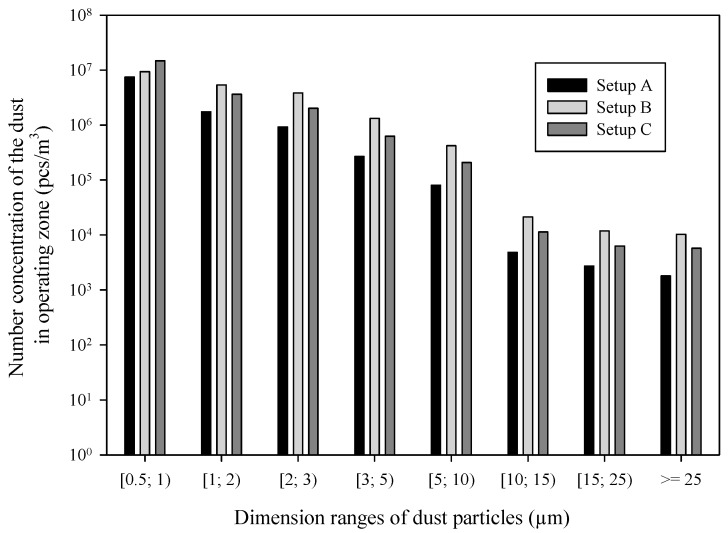
Number concentration of the dust particles in the air surrounding the sawing machine.

The results of dust particle concentration in eight size ranges used in the experiments are shown in Figure 2. In the air surrounding the sawing machine, the smallest number of dust particles in all the dimensional ranges was observed when the setup A of the dust extraction system was applied. In this setup, the total extracted air volume rate exceeded 900 m^3^/h, which was sufficient to maintain good air purity. The largest number of particles was observed in relation to the smallest particles (<1 µm), in setup C. The extraction of air by the pipe disconnected from the upper auxiliary dust suction connector (Setup B) caused a significant reduction of the air flow in the main suction connector, down to the level of 630 m^3^/h. Additionally, no dust capturing had taken place in the upper hood covering the saw. This turned out to be insufficient, and caused the highest content of dust particles in the air compared to other setups of the dust extraction system for almost all dimensional ranges. In general, the number concentration always decreases significantly when particle size grows.

**Figure 3:**
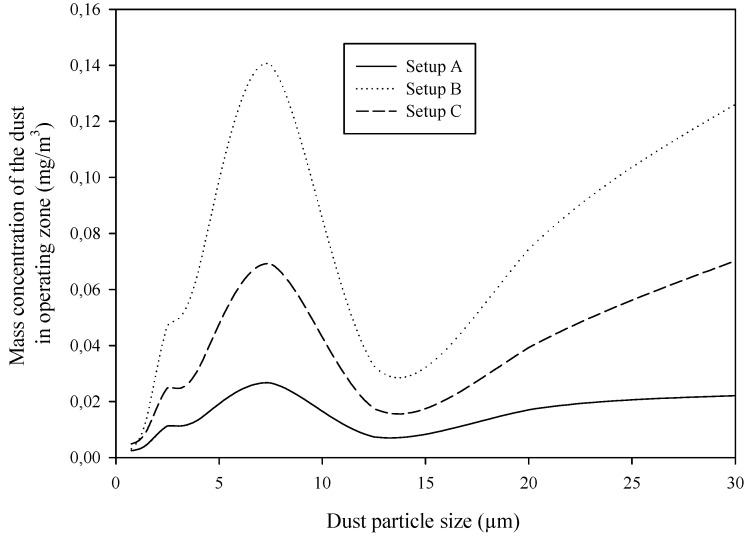
Mass concentration of dust particles in the air at the operating stand.

For the mass concentration, this is no longer true. Figure 3, for clarity, shows a curve representation of mass concentration histograms. All three setups provide similar curves. In all cases, the highest pollution is caused by particles in the range of 5 to 10 µm and those over 25 µm; for health effect reasons, the first group is more dangerous. The particles <10 µm can penetrate the human respiratory tract beyond the larynx and create a thoracic fraction [26]. Setup B remains the worst case, as it was in the number concentration analysis.

**Figure 4:**
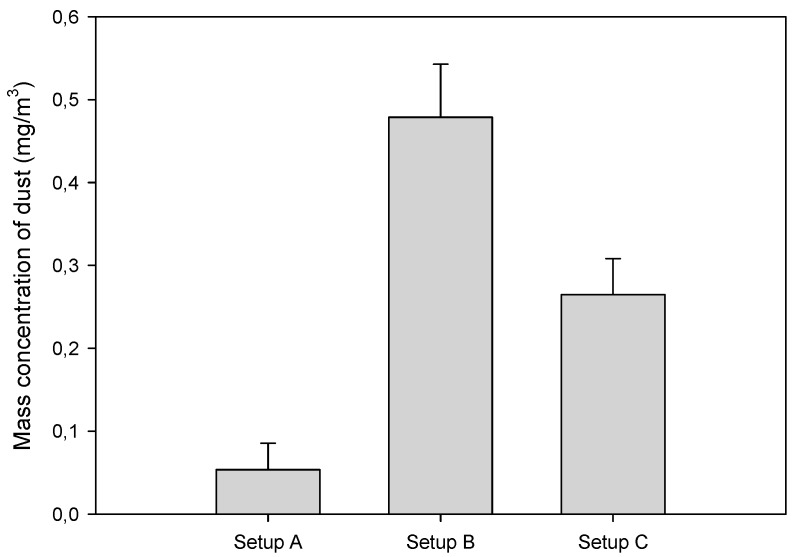
Average mass concentration of dust in the air surrounding the sawing machine during particleboard machining, calculated according to Equation (1) (error bars depicted standard deviation).

Figure 4 shows the total dust mass concentrations for all three setups. There is a significant effect from connecting pipes of the extraction system to the suction connectors of the saw machine on the surrounding air dustiness. The default setup (A), with both upper and lower hoods connected to the exhaust system, caused the lowest mass concentration (0.103 mg/m^3^). It was proven that connecting only one pipe to the bottom connector (setup C) emitted over twice as much dust as the default setting, while setup B was shown to be the most air polluting case, with an increase in total dust mass concentration of about five times in the working zone. Despite the significant differences in this respect between individual setups, the dust concentration values were below 0.5 mg/m^3^. Fujimoto et al. [2] observed significantly higher levels of air dust when sawing particleboard. They achieved 1.13 and 2.84 mg/m^3^ for rotational speeds of the saw of 2000 and 3000 rpm, respectively, with feed per tooth at 0.05 mm. It is known [27,28] that the use of low feed-per-tooth values always creates waste, with a higher content of fine dust fraction. Since in present work, a twice as small feed per tooth was utilized, as in the research of Fujimoto et al. [2], we could expect higher values of dust mass concentration. This phenomenon may be explained by the fact that Fujimoto et al. did not used an upper saw hood. One may then come to the conclusion that the upper hood, even when not connected to the sawdust extraction system, closes the upper, over-the-table half-space and stops, at least partially, dust from spreading in a working zone. 

Black et al. [29] indicate that the high level of dustiness at circular saws in various types of woodworking plants requires special attention devoted to the proper determination of processing parameters, as well as to the effective operation of dust extraction system. In addition to determining the correct rotational speed of the tool and the feed per tooth or chip thickness, appropriate construction and assembly of the upper suction hood is also required. Studies of Barański et al. [30], Barański and Pikała [31], and Barański et al. [32] indicate that improving the design of the upper suction hood can be an effective factor in improving the efficiency of dust extraction from circular saws. In addition, Mikkelsen et al. [33] claim that bad design of the exhaust hood is frequently the reason for increasing dust concentration near woodworking machine.

**Figure 5:**
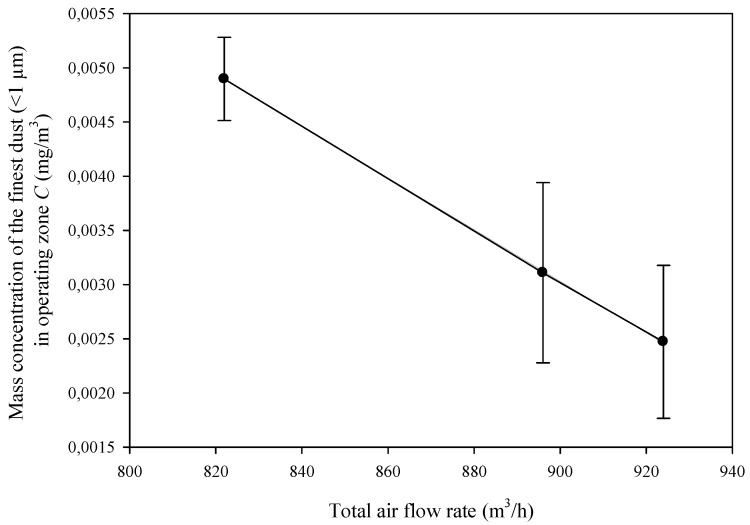
Mass concentration of the finest dust particles in the air at the operating stand (error bars depicted standard deviation).

Kos et al. [7], in their research, concluded that the exhaust system has a great influence on the total wood dust concentration in the surrounding air of woodworking machines, but less impact on respirable particles concentration. The first statement is confirmed by current research, which is clearly visible from the Figure 5 and has been already discussed. When respirable particle concentration is considered in current examinations, the exhaust setup definitely plays an important role in air pollution with dust. Figure 5 presents the mass concentration of the finest dust for the three setups considered. The highest concentration, with a flow of 822 m^3^/h, came from setup C (Table 2), while the lowest was achieved with setup A. This relation is strongly linearly related to the total flow rate of the air being extracted from the surroundings of the machine tool, no matter of numbers of pipes connected or the air flow rates in single pipes. This phenomenon might be explained by the fact that the smallest and therefore the lightest particles are more easily caught from the surroundings of the machine tool than heavier ones. Perhaps when changing the air flow rate in different setups, one will obtain these results on the same regression line? This could confirm the second statement of Kos et al. [7], but it was not the object of current study.

## 4. Conclusions

Based on the results of the measurements of the content of dust particles in the air surrounding the circular saw, it was found that the level of air dustiness highly depends on the setup of the dust extraction system. The upper hood is a crucial element of the local exhaust ventilation system, and its proper construction and connection have a fundamental meaning. The setups included in the current examination differed in the way the upper suction hood was connected to the extraction pipe. Disconnecting the pipe from the upper cover (setup B) significantly increased the air dustiness up to 0.5 mg/m^3^, due to the reduction in the general efficiency of the extraction system. In addition, leaving the air flow in the pipe disconnected from the upper suction hood further degraded the efficiency of the extraction system. The most preferred setup, setup A, consisting of upper and lower hoods connected together to the air exhaust, had a total mass concentration of dust that was five times lower.

Mass concentration of the smallest dust particles has a negative linear relation to the total air flow rate of the exhaust system. Therefore, the care for the proper operation of the dust exhaust system is very important. This prevents the dispersion of dust in the air—i.e., removes the primary cause of dustiness. The use of personal protective equipment, general ventilation, etc., is less effective, because it eliminates the effects of dust dispersion. It is therefore necessary to educate on the principles of design and operation of dust exhaust systems in the wood industry, in order to properly use their capabilities.

## Figures and Tables

**Table 1:** Setup cases of dust extraction system.

Dust Extraction Connector	Setup A	Setup B	Setup C
Main dust extraction connector (120 mm)	connected	connected	connected
Auxiliary dust extraction connector (80 mm)	connected	Disconnected (maintained air flow in the pipe)	Disconnected (shut air flow in the pipe)

**Table 2:** Operation parameters of the dust extraction system.

Dust Extraction Connector	Setup A		Setup B		Setup C	
	Flow Rate *V* (m^3^/h)					
	Required *	Current **	Required	Current	Required	Current
Main dust extraction connector (120 mm)	814	708	814	630	814	822
Auxiliary dust extraction connector (80 mm)	362	216	362	264	–	–
Total	1176	924	1176	896	814	822

* Recommended by the machine manufacturer in the manual to ensure proper sawdust extraction [25]; ** the actual airflow during measurements.
